# Deep brain stimulation and genetic variability in Parkinson’s disease: a review of the literature

**DOI:** 10.1038/s41531-019-0091-7

**Published:** 2019-09-06

**Authors:** Johanne Ligaard, Julia Sannæs, Lasse Pihlstrøm

**Affiliations:** 1 Faculty of Medicine, University of Oslo, Oslo, Norway; 2 Department of Neurology, Oslo University Hospital, Oslo, Norway

**Keywords:** Parkinson's disease, Genetic association study

## Abstract

Deep brain stimulation is offered as symptomatic treatment in advanced Parkinson’s disease, depending on a clinical assessment of the individual patient’s risk-benefit profile. Genetics contribute to phenotypic variability in Parkinson’s disease, suggesting that genetic testing could have clinical relevance for personalized therapy. Aiming to review current evidence linking genetic variation to deep brain stimulation treatment and outcomes in Parkinson’s disease we performed systematic searches in the Embase and PubMed databases to identify relevant publications and summarized the findings. We identified 39 publications of interest. Genetic screening studies indicate that monogenic forms of Parkinson’s disease and high-risk variants of *GBA* may be more common in cohorts treated with deep brain stimulation. Studies assessing deep brain stimulation outcomes in patients carrying mutations in specific genes are limited in size. There are reports suggesting that the phenotype associated with parkin mutations could be suitable for early surgery. In patients with *LRRK2* mutations, outcomes of deep brain stimulation seem at least as good as in mutation-negative patients, whereas less favorable outcomes are seen in patients carrying mutations in *GBA*. Careful assessment of clinical symptoms remains the primary basis for clinical decisions associated with deep brain stimulation surgery in Parkinson’s disease, although genetic information could arguably be taken into account in special cases. Current evidence is scarce, but highlights a promising development where genetic profiling may be increasingly relevant for clinicians tailoring personalized medical or surgical therapy to Parkinson’s disease patients.

## Introduction

Parkinson’s disease (PD) is a neurodegenerative disorder clinically characterized by bradykinesia, tremor, rigidity, and postural instability, as well as a range of non-motor symptoms including cognitive decline and dementia.^1^ Deep brain stimulation (DBS) is currently well established as an adjunct therapy in PD patients experiencing either motor complications not controlled by best medical therapy or medication-refractory tremor. However, as DBS is ineffective against a number of PD symptoms and carries potential risks and side effects, careful individualized patient screening and target selection are essential for good surgical outcomes.

DBS targeting the subthalamic nucleus (STN) and globus pallidus pars interna (GPi) are both effective in reducing motor fluctuations in PD.^2^ The decision to offer surgery should be based on an individual assessment of the risk-benefit profile. The ideal candidates have age below 70–75 and a good levodopa response.^3^ Conversely, DBS is not suitable for patients with predominantly axial symptoms, cognitive impairment or active depression. Current evidence favors STN as the most effective target, although GPi might be considered in patients with pronounced dyskinesias or mild cognitive impairment. DBS targeting the ventral intermediate nucleus of the thalamus (VIM) is an option in elderly PD patients with medication-refractory tremor as the predominant symptom.^4^

Over the last two decades a number of genes causing Mendelian forms of PD have been identified. It has been estimated that Mendelian PD overall accounts for 5–10% of cases, with numbers varying significantly across populations.^5^ For the common, sporadic form of PD, more than 40 genetic risk-loci have been identified through genome-wide association studies (GWAS).^6^ Rare and low-frequency missense variants in *GBA* are strong risk factors for PD, representing an intermediate between Mendelian genes and typical GWAS loci in terms of frequency and effect size.^7^ Evidence indicates that genetic variants causing or conferring susceptibility to PD also show correlations with clinical phenotype, contributing to the striking clinical variability observed across individual PD patients.^8–11^

Given that genetic background partly determines PD phenotype it could plausibly be hypothesized that genetic profiling could be used to predict DBS outcome and help clinicians select the right patients for surgery. Ideally, genetics could provide a rationale for implantation at an earlier stage in subgroups of patients particularly well suited for DBS, and warrant caution in others, where risks and side effects are likely to outweigh the clinical benefit. Such a development would be in line with the principles of personalized medicine or precision medicine, where individualized treatment attuned to the patient’s genetic profile has been proposed as a key element.^12^

In recent years, an increasing number of publications have reported details of DBS treatment and outcomes in genetically characterized PD patients. We present a systematic review of this literature, summarize key insights and discuss the rationale for genetic screening as a clinical tool for patient selection to DBS in PD.

## Results

The 39 articles identified reported studies employing principally two kinds of study design. A few studies performed genetic screening in a group of PD patients treated with DBS comparing the frequency of specific genetic variants against a group not treated with DBS. The majority of studies evaluated the efficacy of DBS in patients of a known genotype, either descriptively or compared quantitatively to a mutation-negative group. Some articles describe a combination of these study designs. Reports of few or single cases constituted about half of the included publications.

The majority of the publications included patients who underwent DBS surgery with implantation in STN. However, a few reported patients with severe troublesome dyskinesia had GPi implantation.^13–20^ Only one mutation-positive patient (*GBA*) was treated with VIM stimulation.^20^ A number of different outcome measures are used to assess DBS treatment response and safety; the most commonly used being the Unified Parkinson’s Disease Rating Scale (UPDRS) and levodopa equivalent daily dose (LEDD) reduction.

### Genetic screening of DBS cohorts

Receiving a DBS implant represents a milestone in the clinical course of PD, which could also be seen as a marker of a particular endophenotype. The predisposition to developing this endophenotype is likely shaped by genetics, implying that relevant genetic variants should be enriched in DBS-treated cohorts. We found three studies comparing the fraction of mutation carriers in a large group of PD patients to a non-DBS group.

Pal et al. screened for parkin, *LRRK2* and *GBA* mutations in young onset PD patients, including 99 receiving DBS treatment and 684 without DBS.^21^ When analyzed together, mutation carriers were significantly more common in the DBS group compared to the non-DBS group (26.5% vs. 16,8%). This enrichment did not reach statistical significance for any individual gene, although a slightly higher rate of carriers was observed for all three genes investigated.

Performing genetic screening in a cohort of 94 DBS-treated PD patients, Angeli et al. identified parkin, *LRRK2* or *GBA* mutations in 29%.^20^ No mutations were found in *SNCA*, *PINK1* or *DJ-1*. No non-DBS control group was included in the study, but comparing with published reports the authors state that the carrier frequency of 29% was much greater than in population-representative cohorts of PD. Interestingly, both these studies highlighted that parkin mutation carriers had earlier disease onset, yet longer disease duration at the time of DBS. In contrast, GBA carriers, who are known to progress faster, had DBS earlier in the disease course.

A study by Johansen et al. reported on genetic screening in 60 DBS-treated and 570 non-DBS PD patients consecutively enrolled in a movement disorder centre.^19^ All patients were screened for *LRRK2* and *SNCA* mutations, whereas investigation of parkin, *PINK1*, *GBA* and other genes was performed depending on age at onset and family history. PD patients carrying mutations in *LRRK2* or parkin were significantly overrepresented in the group who underwent STN-stimulation. Interestingly, this study also included 21 patients receiving VIM stimulation for levodopa-refractory tremor. No mutation was identified in any of these patients.

The two latter studies also compared the postoperative clinical outcomes of mutation-positive patients to that of non-carriers, concluding that no significant differences can be detected when mutations in all investigated genes are lumped together.^19,20^ Findings relating to specific genes are summarized in the following sections.

### Studies assessing DBS efficacy in monogenic Parkinsonism

#### LRRK2

The most frequent form of monogenic PD is caused by autosomal dominant mutations in *LRRK2*, encoding leucine-rich repeat kinase-2^22,23^, of which the G2019S mutation is the most common.^24^ The normal function of the lrrk2 protein is incompletely understood, but current evidence indicates a role in pathways relating to vesicular transport and lysosomal degradation. The phenotype is similar to idiopathic PD with evidence indicating a slightly more benign course of disease with good response to levodopa, relatively slow decline in motor and cognitive functions and a low burden of non-motor symptoms.^25–27^ We found 11 studies assessing the efficacy of DBS in *LRRK2* mutation carriers. Five of these had sufficient sample size to perform group-wise comparisons of the motor improvement in *LRRK2* PD versus idiopathic PD, while the rest were descriptive studies of smaller cohorts or case reports.

Two studies with similarly sized mutation carrier groups have used statistical tests to compare measures of DBS efficacy, both reporting no significant differences between *LRRK2* carriers and idiopathic PD. A study in Ashkenazi Jewish patients followed 13 G2019S positive PD patients and 26 matched non-carriers for three years postoperatively and assessed the impact of the mutation on treatment outcome in a linear mixed model.^28^ No significant difference was observed in UPDRS off medication on stimulation or LEDD reduction across the two groups. Similarly, relative improvement of UPDRS II-IV and LEDD reduction were not significantly different after 6–12 months in a French study comparing nine *LRRK2*-PD patients, mainly G2019S carriers, to 60 idiopathic PD patients using a *t*-test.^29^ The UK screening study mentioned above also reported that differences in outcome were statistically non-significant, yet this study included only five *LRRK2* patients.^20^

A claim that LRRK2 G2019S patients have greater improvement following surgery for STN-DBS than idiopathic patients was made in an Algerian comparative study of 15 mutation carriers and 12 non-carriers with two years’ follow-up.^30^ UPDRS III improvement the medication off-state was reported at 51.1% in mutation-positive patients versus 25.5% in non-carriers, and similar differences were seen for Hoehn & Yahr and Schwab & England scales, yet without formal statistical testing across groups. Conversely, a study including four *LRRK2* R1441G PD patients in the Basque Country of Spain stated that these had a limited DBS response on motor function, daily life activities and quality of life, an inferior outcome compared to 41 *LRRK2* mutation-negative DBS-treated control patients, yet not supported by statistical hypothesis testing.^31^

Several reports of few or single cases have also documented sustained improvement after DBS in PD caused by different *LRRK2* mutations (Table 1), with follow-up up to eight years.^32,33^ A few report a beneficial effect, but highlight challenges in managing dystonia or dyskinesias postoperatively.^34,35^ A comprehensive 2008 assessment of *LRRK2*-positive PD identified 12 DBS cases and descriptively reported good or excellent clinical outcome in eight of the patients, moderate in two and poor outcome in the last two patients.^26^ In a single case report of a *LRRK2* N1437H-carrier with significant psychiatric comorbidity, bradykinesia, rigidity and dystonia deteriorated a few weeks postoperatively and the patient committed suicide six months after the surgery.^36^

#### Parkin

The most common form of autosomal recessive PD is caused by loss of function mutations in *PARK2*, encoding the parkin protein, probably explaining 1–8% of early-onset PD cases.^37^ Parkin is a component of the ubiquitin-proteasome system and binds to the membrane of damaged mitochondria selected for degradation through mitophagy. The phenotype is characterized by early onset and a predominantly motor syndrome with dystonia, freezing of gait and early fluctuations, yet slow progression and relatively little cognitive or autonomic involvement.^5^ This indicates that parkin patients may be good candidates for DBS treatment.

We identified four studies performing group-wise statistical analyses of DBS outcome measures comparing parkin mutation carriers to non-parkin PD. A French study included 54 patients with early-onset PD treated with STN-DBS, out of which seven had biallelic parkin mutations (homozygous or compound heterozygous) and seven had one identified mutation.^38^ Twelve months postoperatively, patients with biallelic mutations had significantly lower LEDD than mutation-negative patients. Performance on the Mattis dementia scale was significantly lower in the parkin group on follow-up only, but the authors state that this could possibly also reflect a significantly longer disease duration at the time of surgery. A Korean study reported earlier age at onset and longer disease duration at DBS surgery, but no significant difference in postoperative outcomes comparing three homozygous or compound heterozygous parkin patients to nine mutation-negative early-onset PD patients.^39^ A similar result was found in a UK screening study that identified four DBS-treated parkin double mutation carriers.^20^ An Italian study found no statistically significant differences between five parkin mutation carriers and 31 mutation-negative patients.^40^ In this study however, only one patient in the parkin group had two identified mutations. A German-Canadian publication descriptively compared a mutation carrier group of one *PINK1* and 11 parkin patients to 68 mutation-negative patients.^41^ The study highlighted more pronounced axial symptoms in the mutation group both before and early after surgery, which was evened out at 3–6 years postoperative follow-up.

We identified seven further reports of one or a few parkin PD patients receiving DBS treatment, most of which highlight a good outcome.^14,42,43^ In particular, there are reports of DBS efficacy after extremely long disease durations (up to 45 years)^44,45^, and a sustained response many years postoperatively.^13^ A few reported parkin patients had electrodes implanted in GPi mainly to control dyskinesias, one even as a second target after several years of STN-DBS.^14^ A publication reporting long-term follow-up in Arabic parkin kindreds emphasize that only modest improvement was seen after DBS in three patients where axial symptoms were prominent.^46^

#### Other Mendelian genes

Oligomerization and aggregation of alpha-synuclein plays a pivotal role in PD pathogenesis, and the protein forms the main component of the neuropathological hallmark, Lewy bodies. Point mutations^47^ and genomic multiplications^48^ involving *SNCA* are rare causes of autosomal dominant PD. Triplication is associated with a severe form of PD with early-onset, rapid progression and dementia whereas patients with duplications may resemble idiopathic PD.^49^ We identified two case reports, each describing a patient with *SNCA* duplication treated with STN-DBS. Both reported a good response with substantial improvement of UPDRS-III scores and reduction of LEDD postoperatively, and only slight to moderate decline in cognitive function after one and 3 years, respectively, in line with overall STN-DBS results in PD.^50,51^ A third case report describe a patient with a mosaicism of *SNCA* duplication show an overall good outcome of DBS, where GPi was chosen as target due to prominent dyskinesias and mild cognitive impairment.^15^

Mutations in *VPS35* cause autosomal PD with a phenotype that is clinically indistinguishable from idiopathic PD.^52,53^ We identified a total of five articles, all describing PD-patients with the D620N *VPS35* mutation treated with DBS. As part of screening efforts following the identification of the gene, two mutation carriers treated with DBS were reported, one with a good motor outcome^54^, the other implanted at high age with a small benefit, yet complicated by dysarthria.^55^ Two publications have followed-up kindreds included in one of the studies that originally linked the gene to PD, reporting DBS treatment in one US^56^ and two Swiss patients^57^ with a good response, sustained for up to eight years postoperatively. Similarly, an excellent long-term motor response to DBS was described in a Taiwanese patient carrying the *VPS35* D620N mutation.^58^

*PINK1* encodes PTEN-induced putative kinase 1, which has a role in mitochondrial quality control forming protein-protein interactions with parkin. Similar to parkin, *PINK1* mutations cause autosomal recessive PD that is clinically characterized by early onset, slow progression and a good response to dopaminergic treatment. One patient with homozygous *PINK1* mutation was reported together with parkin mutations in a German-Canadian study mentioned above.^41^ Apart from this publication, we identified only one article showing the DBS outcome of a homozygous *PINK1* mutation carrier, highlighting successful treatment with GPi-DBS in a patient with prominent dystonia and dyskinesias.^16^

### Genetic risk variants as predictors of DBS outcome

#### GBA

This gene encodes the lysosomal enzyme alpha-glucocerebrosidase. Homozygous mutations in this gene cause Gaucher’s disease, an autosomal recessive lysosomal storage disorder where Parkinsonism occurs in a subset of patients. In the heterozygous state, however, the same pathogenic variants are strong risk factors for PD, yet without sufficient penetrance to cause a Mendelian inheritance pattern.^59^ Different *GBA* mutations show a spectrum of severity with respect to impact on enzyme activity and effect on PD susceptibility, ranging from around tenfold increased risk for carriers of L444P^60^ to less than twofold in the low-frequency variant E326K,^61,62^ which does not cause Gaucher’s disease in the homozygous state. It is now well established that *GBA* mutations are associated with a more severe PD phenotype characterized by early onset, rapid motor progression, more prominent cognitive decline and a high burden of other non-motor symptoms, with worse outcomes for the most severe mutations.^63–65^ We found five articles reporting on the outcome of DBS treatment in patients with *GBA* mutations.

A study from the UK matched 17 patients with GBA mutations to 17 non-carriers and assessed DBS outcome with a mean postoperative follow-up of 7.5 years.^17^ Motor symptoms, LEDD and stimulation settings did not differ significantly between groups. On long-term follow-up, cognitive decline was more prevalent and more severe in *GBA* mutation carriers, and outcomes were worse for non-motor symptoms and quality of life. The reported results were likely underestimates, as five *GBA* positive patients were lost to follow-up due to severe disability or death. Similar results were also highlighted in an earlier publication from the same group, where cognitive and axial impairment were significantly more pronounced in the group of 15 *GBA* mutation carriers on longitudinal follow-up after GPi or STN-DBS.^20^ A German study identified four DBS-treated PD patients carrying a *GBA* mutation through screening, matched each *GBA* case to two non-carriers and compared clinical data from 4–10 years of follow-up.^66^ Both mutation carriers and non-carriers reduced their LEDD and had well controlled motor fluctuations and dyskinesias postoperatively. However, on long-term follow-up therapy-resistant axial symptoms and cognitive decline were markedly more pronounced in the *GBA* group. A large-scale French *GBA* screening study reported two *GBA* carriers treated successfully with DBS, one of these being homozygote for the N370S mutation.^67^ A retrospective study from a Gaucher’s disease clinic in Israel identified two Gaucher’s disease patients also diagnosed with PD who were treated with DBS with a dramatic and sustained symptomatic improvement.^68^

#### 22q11.2 microdeletion

We found one article describing the 22q11.2 microdeletion, known to increase the risk of PD, in which three of the patients who underwent DBS had a satisfactory improvement of UPDRS-III score with 30–70%.^18^

#### Common, non-coding variants in the SNCA and LRRK2 loci

Large-scale meta-analyses of GWAS in PD have identified an increasing number of common genetic risk variants.^6,69^ Individually, each of these common variants has a small effect on disease susceptibility, and in general, large samples are required for adequate statistical power in genetic association studies of common variants. There is currently increasing evidence showing that common variant risk profile plays a role in shaping PD phenotype, although sample size remains a challenge for association studies of clinical outcomes.^8,9,70^

We identified one study assessing the association between DBS outcome and common low-risk variants from GWAS.^71^ Eighty-five patients who underwent STN-DBS were genotyped for single-nucleotide polymorphisms in the *SNCA* (rs356220) and *LRRK2* (rs1491923) loci. Treatment response was measured as difference in UPDRS III in the stimulation on/medication off-state two years postoperatively versus medication off before surgery. The risk-allele of the *SNCA* variant rs356220 was weakly associated with UPDRS III improvement at *p* = 0.03, but not with baseline scores, indicating that the variant could be a prognostic marker for DBS outcome. No association was seen for the *LRRK2* variant.

## Discussion

Surgical therapy with DBS can alleviate symptoms and improve quality of life in a subset of patients with advanced PD. Where current patient and target selection relies on assessing the individual risk-benefit profile through careful clinical screening, future DBS considerations could potentially incorporate genetic information, in line with the principle of personalized medicine. To summarize the current evidence on genetic variability and DBS treatment and outcomes in PD, we performed a systematic review of the literature, identifying 39 publications of interest. Overall, the numbers of included patients are small and the methodology is heterogeneous across studies, providing scarce evidence for any firm conclusions.

Studies based on genetic screening of patient cohorts comparing DBS-treated PD to patients not receiving DBS indicate that both certain forms of monogenic PD (*LRRK2* and parkin) and carriers of the strong *GBA* risk alleles may be overrepresented in the DBS group. This is in line with the observation that these genes predispose to particular phenotypic profiles that to some extent overlap with clinical criteria for DBS eligibility. Interestingly, no enrichment of genetic forms of PD was reported in patients receiving VIM-DBS for treatment-refractory tremor, indicating that genetics may be less clinically relevant for this group, compared to DBS candidates with motor fluctuations.^19^

When DBS electrodes are implanted and the stimulation parameters established, we expect a therapeutic response on a subset of motor symptoms. Overall, the evidence gives no reason to believe that this immediate postoperative effect should be any different in patients with a specific genotype. Consequently, the potential clinical relevance of genetic information would relate to its impact on *prognosis*. DBS prognosis, in turn, depends largely on whether fluctuations of dopa-responsive motor symptoms remain predominant, or are outweighed by non-motor and axial symptoms. For the three most studied genes, parkin, *LRRK2* and *GBA*, the current literature gives some indication about DBS outcomes on a group level.

The PD phenotype caused by parkin mutations corresponds well with the prototypic DBS patient, with early onset of a largely pure motor syndrome and pronounced motor fluctuations, yet minimal cognitive decline, even after many years of disease. Several reports document good response to DBS despite long disease duration both before and after surgery in parkin patients. It could be argued that a status with biallelic parkin mutations would weigh somewhat in favor of surgery, probably sooner rather than later, in a case where DBS treatment is considered.

Carriers of *LRRK2* mutations generally have a phenotype similar to idiopathic PD, possibly with a slightly more benign disease course. In line with this, the largest studies of DBS in *LRRK2* patients show no significant differences in outcome compared to non-carriers.^28,29^ Some conflicting evidence is presented in small studies and case reports, but taken together we see no current rationale for *LRRK2* status to impact clinical considerations concerning DBS treatment in PD.

Heterozygous *GBA* mutations are associated with a severe phenotype and fast progression of both motor and non-motor symptoms. Several studies show that these patients tend to require DBS early in the disease course because of rapid motor progression.^17,20,21^ Furthermore, current evidence suggests that *GBA* carriers show faster cognitive decline and development of axial symptoms following surgery.^66^ The authors of the largest study on *GBA* and DBS conclude that *GBA* status could be an important consideration when weighing the benefits and risks associated with surgery.^17^ It should be emphasized, that even though mutation carriers may have less clinical benefit than non-carriers, this does not imply that there is *no* relevant benefit. A critical question concerns cognitive decline, where some changes are normally seen following DBS, yet no general reduction in overall cognition.^72^ Whether *GBA* carriers are particularly vulnerable to cognitive side effects of DBS, or if worse postoperative outcomes merely represent disease progression in itself, remains to be determined. Some authors argue that *GBA* mutation carrier status could weigh in favor of targeting GPi, which is known to have less impact on cognition than STN-DBS.^17^

It is worth noting that a positive association between genotype and DBS outcome, as reported in a number of publications reviewed here, does not necessarily imply that genetic testing is clinically helpful. To definitely prove its independent utility, studies would have to show that genetic testing gives an added prognostic value, over and above what can already be obtained through careful clinical assessment when the patient is evaluated for DBS. Large prospective cohorts combining genetic profiling with deep phenotyping may provide suitable data to address this question.

## Conclusion

Careful assessment of clinical symptoms remains the dominant basis for clinical decisions associated with DBS surgery in PD. We believe however, that weighing in genetic information could probably be justified in very special cases. In the future, we anticipate that our ability to predict PD subtype based on genetic profiling will increase substantially, and that genetic information will be one important resource among many for clinicians tailoring individualized medical or surgical therapy to PD patients. Although this review has emphasized coding variants in single genes, one small article points towards the possibility of genetic profiling based also on common risk variants.^71^ Polygenic scores capturing the cumulative effect of multiple common variants could ultimately make the genetic background clinically relevant also for the many PD patients falling into the “mutation-negative” category in the majority of studies reviewed here.

## Methods

**Fig. 1:**
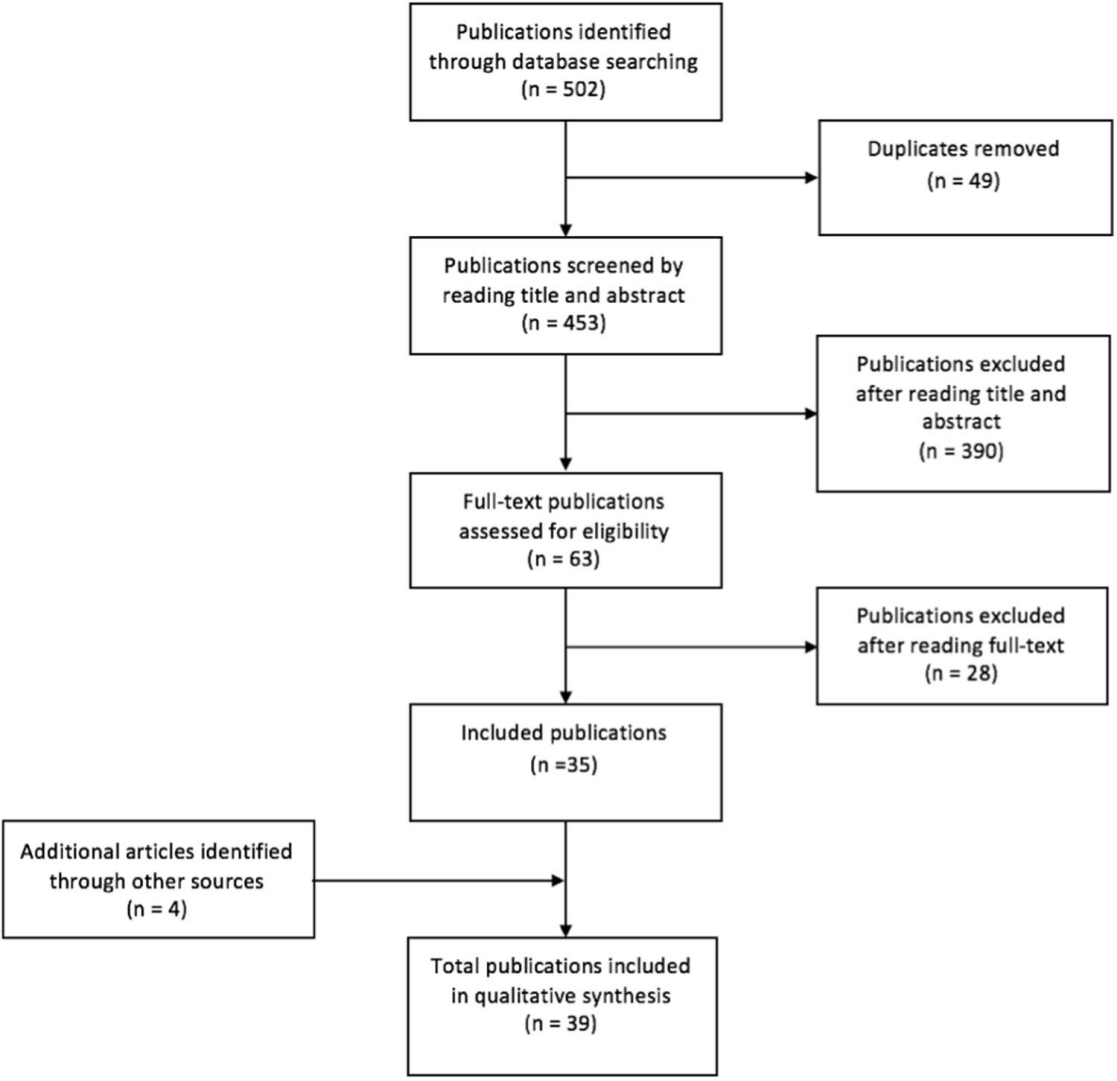
Overview of the search strategy. The flowchart shows how publications were identified and screened to arrive at the final 39 articles included in the review

We defined publications of interest as articles reporting patient series or cases characterized by both genetic status and DBS outcome, meaning either DBS treatment versus no DBS as a clinical outcome in itself, or any measure of DBS efficacy or safety. The strategy of the systematic search is shown in Fig. 1. A Medical Subject Heading (MeSH)/emtree-based search in The Embase database was performed using the following search terms: ((Parkinson disease OR Parkinson* disease) AND (brain depth stimulation OR deep brain stimulat* OR DBS) AND (genotype OR genetic heterogeneity OR genetic variability OR gene OR genes OR genetic* OR genotyp* OR heterogeneity OR mutation*)). The search was set to include full text articles and letters, but excluded conference abstracts, editorials and notes. In order to capture articles in process still awaiting MeSH-term indexing, we repeated the search in PubMed with identical search terms, only unlinked to MeSH index. The searches were performed on 16 January 16 2018, identifying 453 publications out of which 390 were excluded by reading the title and abstract. The remaining 63 publications were read in full by both first authors. Cases of diverging opinions about an article’s relevance were discussed by all authors, leaving 35 publications of interest. By screening of reference lists, four additional articles were identified, adding up to a total number of 39 publications included in our review (Table 1).

**Table 1:** Overview of published articles reporting on genetics and deep brain stimulation in Parkinson’s disease

Reference	Study design	Material	Follow-up	Main finding
Pal et al.^21^	Genetic screening of DBS vs non-DBS EOPD cohorts with assessment of predictors for DBS treatment	98 DBS-treated EOPD vs 654 non-DBS (US)	Cross-sectional	PD with either *GBA* (12,1% vs 8,0%), *LRRK2* (5,1% vs 3,9%) or parkin (10,2% vs 6,1%) mutations were significantly more common in DBS group compared with the non-DBS group (26,5% vs 16,8%).
Angeli et al.^20^	Genetic screening of DBS cohort with assessment of DBS outcomes in MC vs iPD	94 DBS-treated PD (UK)	Cross-sectional	PD with either *GBA* (17,0%), *LRRK2* (5,3%) or parkin (8,5%) mutations were significantly more common in DBS group (29%) compared to population-representative cohorts of PD.
			1 year (+5years)	No significant difference in improvement of UPDRS III score between mutation carrier groups and non-carriers postoperatively. *GBA* mutation carriers had a larger cognitive decline compared to non-carriers at five year follow-up.
Johansen et al.^19^	Genetic screening of DBS vs non-DBS cohorts with assessment of DBS outcomes in MC vs iPD	60 DBS-treated PD vs 570 non-DBS (Norway)	Cross-sectional	PD with either *LRRK2* (5% vs 0,9%) or parkin (16,7% vs 3,1%) mutations were more common in the DBS group compared with the non-DBS group. No difference in the proportion of *PINK1*-carriers in the two groups.
			1, 3, and 5 years	No significant differences in DBS outcome between mutation carriers, regardless of mutation type, and non-carriers looking at clinical features at baseline compared to follow-up.
Greenbaum et al.^28^	Statistical comparison of DBS outcomes in MC vs iPD	13 *LRRK2* G2019S PD vs control group of 26 iPD (Israel)	6–12 months + 3 years	Significant improvement of UPDRS-III score and LEDD in both groups, but no significant difference between MC and iPD at baseline or at follow-up.
Schupbach et al.^29^	Statistical comparison of DBS outcomes in MC vs iPD	8 *LRRK2* G2019S PD and 1 *LRRK2* T2032S PD vs 60 iPD (France)	6–12 months	No significant difference in improvement of UPDRS II-IV or LEDD reduction between the two groups.
Sayad et al.^30^	Descriptive comparison of DBS outcomes in MC vs iPD	15 *LRRK2* G2019S PD vs control group of 12 iPD (Algerie)	2 years	More pronounced postoperative improvement of UPDRS III off-medication (51.1% vs 25.5%), S&E and H&Y with DBS in *LRRK2*-group.
Gomez-Esteban et al.^31^	Descriptive comparison of DBS outcomes in MC vs iPD	4 *LRRK2* R1441G PD vs 41 iPD (Basque country, Spain)	6 months	Less improvement of UPDRS II-III scores and quality of life in MC compared to iPD.
Lesage et al.^32^	Descriptive case series	2 *LRRK2* G2019S PD (France) 1 *LRRK2* T2031S PD (Spain)	7 years	Sustained long-term improvement in UPDRS III.
Breit et al.^33^	Descriptive case report	1 *LRRK2* R793M PD (Germany)	1 + 8 years	Sustained long-term improvement in UPDRS III and reduction in LEDD.
Perju-dumbrava et al.^34^	Descriptive case report	1 *LRRK2* Y1699C PD (Australia)	2/6 weeks + 2,5 years	Sustained improvement in UPDRS-III and sustained reduction >50% in LEDD, but a mild increase in dyskinesia.
Stefani et al.^35^	Descriptive case report	1 *LRRK2* G2019S PD (Italy)	1 + 3 months	Improvement of UPDRS-II and UPDRS-III with DBS, but sensitive to levodopa-induced dyskinesia.
Healy et al.^26^	Descriptive case series	12 *LRRK2* G2019S-PD (UK)	Unspecified	Eight patient had a good or excellent outcome, two moderate and two poor.
Puschmann et al.^36^	Descriptive case report	1 *LRRK2* N1437H PD (Sweden)	Few weeks + 6 months	Poor outcome postoperatively, committed suicide after six months.
Lohmann et al.^38^	Statistical comparison of DBS outcomes in MC vs iPD	14 parkin-PD (1 homozygous, 6 compound heterozygous, 7 single heterozygous) vs 39 iPD (France)	12–24 months	Significantly earlier age at onset and longer disease duration in patients with two parkin mutations. Postoperatively no differences in UPDRS III improvement, significantly lower LEDD, lower MATTIS dementia rating scale score and higher Hoehn and Yahr score (non-significant if excluding one case of ballistic dyskinesia) in double mutation carriers.
Kim et al.^39^	Statistical comparison of DBS outcomes in MC vs iPD	3 homozygous/compound heterozygous parkin-PD vs 9 iPD (South Korea)	2 years	Significantly younger AAO and significantly longer disease duration before surgery in MC than NC.
			5 years	No significant difference in improvement of UPDRS-II-, UPDRS-III-score or H&Y between the two groups.
Romito et al.^40^	Statistical comparison of DBS outcomes in MC vs iPD	5 Parkin-PD (1 compound heterozygous, 4 single heterozygous) 31 iPD (Italy)	21.6 ± 13.1 months	No difference in mean UPDRS III improvement on stim-off-med (56% vs 51%). LEDD reduced by a higher degree in the parkin group, but not statistically significant (76% vs 55%).
Moro et al.^41^	Descriptive comparison of DBS outcomes in MC vs iPD	11 parkin-PD (4 homozygous, 2 compound heterozygous, 5 single heterozygous) + 1 homozygous PINK1-PD vs 68 iPD (Canada/Germany)	Short-term: 3–12 months Long term: 3–6 years	In short-term follow-up less UPDRS improvement (36% versus 56%) and higher burden of axial symptoms in MC. No difference in long-term follow-up (UPDRS III improvement on stim-off-med vs off-med preoperatively 42% vs 44%,).
Capecci et al.^42^	Descriptive aase report	1 homozygous parkin-PD (Italy)	12 months	UPDRS III score improved by 84%, LEDD reduced by 66%, severe dyskinesias disappeared. Improvement in both PDQ-39, BDI and BDS measures.
Isaacs et al.^14^	Descriptive case report	1 compound heterozygous Parkin-PD (USA)	Several years	1st DBS (STN): several years of significant motoric benefit, but stimulation- and medication-refractory dystonia. 2nd DBS (GPi, due to dystonia): dystonic symptoms only mildly improved.
Moll et al.^43^	Descriptive case report	1 compound heterozygous Parkin-PD (Germany)	Unspecified	Improvement of motor symptoms and daytime drowsiness, LEDD reduced.
Nakahara et al.^44^	Descriptive case report	1 homozygous parkin-PD with a co-existing heterozygous *PINK1*-mutation (Japan)	12 days + 8 months	DBS afer 45 years’ disease duration. Improvement of UPDRS I-IV-scores and LEDD-reduction by >50%.
Lefaucheur et al.^45^	Descriptive case report	1 compound heterozygous parkin-PD (France)	6 months	DBS after 44 years’ disease duration. Improvement of UPDRS III and IV by >50%, LEDD reduction by 67% postoperatively and stable cognitive function.
Thompson et al.^13^	Descriptive case series	2 homozygous parkin-PD (USA)	3 + 8 years	GPi-DBS: long-term benefit >8 years (less improvement than patient treated with STN-DBS). STN-DBS: sustained improvement >3 years.
Hassin-Baer et al.^46^	Descriptive case series	3 homozygous parkin-PD (Israel)	Unspecified	Modest improvement in appendicular symptoms, no change in axial features (particularly PIGD and LBP).
Antonini et al.^50^ 2011	Descriptive case report	1 *SNCA*-duplication PD (Italy)	12 months	UPDRS-III score reduced by 64%, LEDD reduced by 50% and depression severity decreased on BDI. Complicated by right foot dystonia and nocturnal akinesia. Postoperative decline in verbal fluency and attention shifting.
Elia et al.^51^	Descriptive case report	1 *SNCA*-duplication PD (Italy)	3 years	UPDRS score improved by 42% and LEDD reduced by 58%. MMSE worsened from 26,3/30 to 23,2/30.
Perandones et al.^15^	Descriptive case report	1 *SNCA*-duplication PD (Argentina)	1 month	Improvement in motor features and reduction in pharmacological treatment.
Sheerin et al.^54^	Descriptive case report	1 *VPS35* D620N-PD (UK)	Unspecified	Good outcome.
Kumar et al.^55^	Descriptive case report	1 *VPS35* D620N-PD (Germany)	Unspecified	Modest effect, complicated by dysarthria.
Sundal et al.^56^	Descriptive case report	1 *VPS35* D620N-PD (USA)	Unspecified	Good outcome.
Fleury et al.^57^	Descriptive case series	2 *VPS35* D620N-PD (Swiss)	1 and 8 year(s)	First patient with 76% improvement of UPDRS III (on/off versus off/off) both 1 and 8 years postoperatively. Second patient with 36% improvement of UPDRS 1 year postoperatively, but increased frequency of freezing episodes and falls.
Chen et al.^58^	Descriptive case report	1 *VPS35* D620N-PD (Taiwan)	5 years	37% improvement UPDRS III-score (off-medication), and decrease of peak-dose dyskinesia.
Borrelini et al.^16^	Descriptive case report	1 *PINK1* L347P-PD (Philippines)	1 + 2 months	No difference in UPDRS III-score on-medication, but reduction in UPDRS IV-score and LEDD postoperatively.
Lythe et al.^17^	Statistical comparison of DBS outcomes in MC vs NC	17 *GBA*-PD vs 17 non-GBA PD (UK)	Mean 7.5 years	Significantly worse cognitive outcome (measured by AMSS) and quality of life assessment (PDQ-39) and significantly greater burden of non-motor symptoms (NMSS) for MC compared to NC. No significant difference in UPDRS-III-scores and LEDD-reduction between the two groups.
Weiss et al.^66^	Descriptive comparison of DBS outcomes in MC vs NC	1 N370S *GBA*-PD + 2 L444P *GBA*-PD vs 6 non-*GBA* PD (Germany)	4 + 8 years	Postoperatively good response on UPDRS III-IV and LEDD reduction in both groups. On long-term follow-up more pronounced therapy-resistant axial symptoms and cognitive decline in *GBA* group.
Lesage et al.^67^	Descriptive case series	1 L422Pfs *GBA*-PD + 1 homozygous N370S *GBA*-PD (France)	2 years	Both patients improved in motor function, with less fluctuations and dyskinesias after surgery, but one patient deteriorated due to postural instability.
Chetrit et al.^68^	Descriptive case series	1 N370S/V394L *GBA*-PD +1 homozygous N370S *GBA*-PD (Israel)	Unspecified	Sustained symptomatic improvement.
Dufournet et al.^18^	Descriptive case series	3 microdeletion 22q11.2-PD (France)	Unspecified	30%–70% improvement of the UPDRS-III score.
Weiss et al.^71^	Genetic association study of common SNPs versus DBS response	85 DBS-treated PD genotyped for rs356219 or rs356220 (*SNCA*), 71 genotyped for rs1491923 (*LRRK2*) (Germany)	2 years	rs356220 significantly associated with DBS response assessed by total UPDRS III as well as for the axial motor outcome. Common risk variant in the *LRRK2* locus not associated.

*AAO* age at onset of PD, *AMSS* Age-Corrected Mayo’s Older Americans Normative Studies (MOANS) Scaled Score, *BDI* Beck Depression Inventory, *BDS* Brown’s Disability Scale, *DBS* deep brain stimulation, *EOPD* early-onset parkinson’s disease, *GPi* Globus Pallidus internus, *H & Y* Hoehn and Yahr stage, *iPD* idiopathic parkinson’s disease, *LBP* lower back pain, *LEDD* Levodopa equivalent daily dose, *MC* mutation carrier, *MMSE* Mini mental state examination, *MDRS* Mattis Dementia Rating Scale, *NC* non carrier, *NMSS* non-motor symptom assessment scale, *iPD* idiopathic Parkinson’s disease, *PD* Parkinson’s disease, *PDQ-39* Parkinson’s Disease Questionnaire, *PIGD* postural instability and gait disorder, *S&E* Schwab and England quality of life scale, *SNP* single-nucleotide polymorphism, *STN* sucthalamic nucleus, *UPDRS* Unified Parkinson’s disease rating scale
